# *Salmonella enterica* isolates from Western Australian rangeland goats remain susceptible to critically important antimicrobials

**DOI:** 10.1038/s41598-018-33220-5

**Published:** 2018-10-17

**Authors:** Khalid Al-Habsi, David Jordan, Ali Harb, Tanya Laird, Rongchang Yang, Mark O’Dea, Caroline Jacobson, David W. Miller, Una Ryan, Sam Abraham

**Affiliations:** 1 School of Veterinary and Life Sciences, Murdoch University, Murdoch, WA 6150 Australia; 2 New South Wales Department of Primary Industries, 1243 Bruxner Highway, Wollongbar, NSW 2477 Australia

## Abstract

This study investigated faecal carriage and antimicrobial resistance (AMR) of *Salmonella enterica* recovered from rangeland goats. Faecal samples (*n* = 400) were collected at slaughter from four consignments of goats (*n* = 100 samples per consignment), each from one of four localities in Western Australia. Carriage of *Salmonella* spp. was detected in 106 samples (26.5%; 95% CI 22.4–31.0%). The rate of faecal carriage for each consignment ranged between 23–30%. PCR assays targeting the STM2755 and STM4497 genes revealed 84.9% (90/106) of the isolates were of serovar Typhimurium. *Salmonella* Chester (11/106, 10.4%) and *S*. Saintpaul (5/106, 4.7%) were characterised at inv*A* and omp*F* genes. Antimicrobial susceptibility testing demonstrated that 84.0% of isolates were susceptible to all tested (*n* = 13) antimicrobials. Resistance was identified to azithromycin (14.2%), tetracycline (10.4%), ampicillin (5.7%), amoxicillin–clavulanate and cefoxitin (3.8%), trimethoprim/sulfamethoxazole (1.9%), gentamicin and streptomycin (0.9%). No isolate was resistant to four or more antimicrobials, or to critically important antimicrobials such as fluoroquinolones and extended spectrum cephalosporins. This is the first study reporting AMR in *Salmonella* isolates from Australian rangeland goats. The rate of detection of AMR was very low, some resistance to low-importance drugs was present in the *Salmonella* population, despite the absence of active selection pressure.

## Introduction

Antimicrobial resistance (AMR) is a human and animal health issue due to the potential for the transmission of antimicrobial resistant bacteria between animals and humans via a number of pathways^1^. There are widespread concerns that emergence of antimicrobial resistance in animal bacteria to last line or so called ‘critically important antimicrobials’ (CIAs) can have detrimental impacts on human health^2^. CIA-resistance in non-typhoidal *Salmonella* is of special interest because the organism is readily transmitted to humans in under-cooked meat products, via direct contact with animals and via the environment^3^. Although most such infections are self-limiting, a small proportion can result in serious disease with poor outcomes when the range of therapeutic options is limited due to the pathogen possessing resistance to CIAs^4^.

Non-typhoidal *Salmonella* that are resistant to CIAs appear to be very rare in Australian food-producing animals^5,6^. This has been attributed to the country’s isolation, quarantine restrictions and extensive livestock production systems. Extensive production systems in rangeland regions are characterised by very low stocking densities, ranging down to approximately one animal per ten hectares on remote grazing properties. Livestock in these production systems are infrequently handled for husbandry procedures, and never housed. Further to this, strong regulations virtually exclude the use of CIAs in Australian food- producing animals^7^. For example, fluoroquinolones cannot be used for food producing animals in Australia. Nevertheless, in other parts of the world carbapenemase-producing Enterobacteriaceae (*Escherichia coli*) have been detected in livestock (buffalo calves and beef cattle)^8^, and recently in free-ranging Australian silver gull (*E*. *coli*)^9^ and domestic cats^10^ (*Salmonella enterica* Typhimurium) have been shown to harbour these organisms despite the low likelihood that any animals in these populations have ever received antimicrobials^9,11^. This raises the possibility that extensively-grazed food-animals in remote areas might also be vulnerable to colonisation with CIA resistant *Salmonella*, even though the chance that such animals are exposed to antimicrobials is extremely low due to the low incidence of bacterial disease, and lack of opportunity to handle animals to administer treatments if required.

The Australian goat-meat industry is dominated by rangeland goats, with approximately 90% of products exported^12^. Rangeland goats are a composite breed naturalised throughout Australian pastoral regions (rangelands). These goats are typically unmanaged (undomesticated) and opportunistically captured and utilised for meat production. A great deal of effort is expended during processing to minimise the transfer of faecal flora (including *Salmonella*) onto carcases^13,14^. A number of enteric pathogens, including *S*. Typhimurium, have been reported in rangeland goats^15–18^. Serotyping and phage-typing of rumen, faecal and carcass samples from rangeland goats at slaughter identified that the predominant *Salmonella* serovars were Saintpaul, Typhimurium and Chester^14^.

Currently there is limited data on the carriage of antimicrobial resistance among rangeland goats. Knowledge of the occurrence of AMR in rangeland goats is useful for understanding the ecology of AMR in general because these animals have negligible contact with humans, are rarely subject to management interventions (including antimicrobial treatment), and are typically stocked at low density in remote, semi-arid and arid areas of Australia. The aims of the present study were to determine the prevalence and antimicrobial resistance status of non-typhoidal *Salmonella* in the faeces of Australian rangeland goats presented for slaughter in Western Australia.

## Materials and Methods

### Study design and sample collection

**Figure 1:**
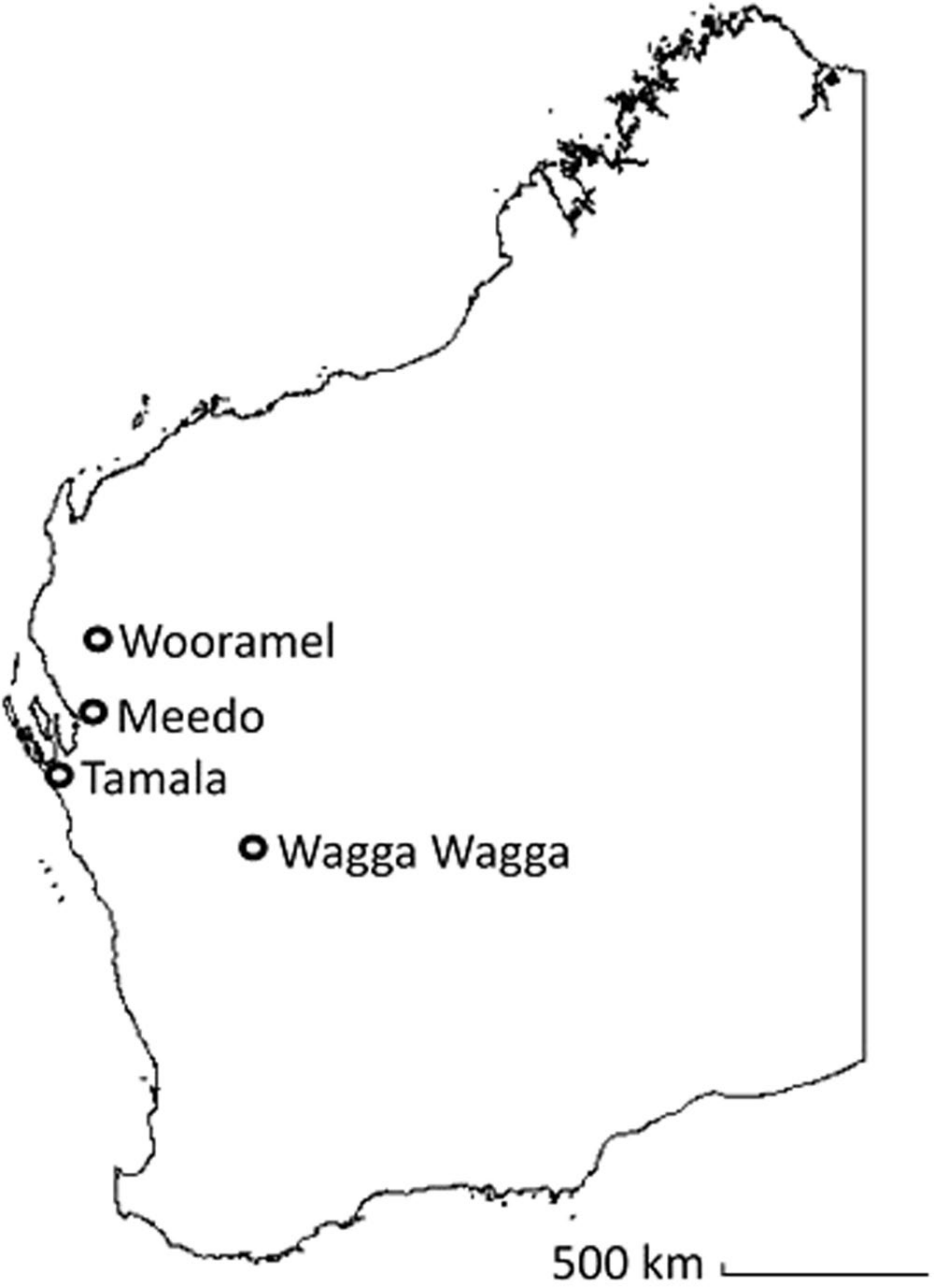
Map depicting locations of the stations from which goat consignments were sourced.

Rangeland goats (*Capra hircus*) were sampled at a processing establishment (abattoir) located in the Beaufort River region of Western Australia that processes goats, sheep and occasionally deer. Goats were captured from rangeland properties in the arid zone of Western Australia. Four separate consignments (each from one rangeland property) were sampled between November and December 2016. Consignments were sourced from Meedo Station in the Carnarvon region, Tamala Station in the Shark Bay region, Wagga Wagga Station in the Yalgoo region, and Wooramel Station in the Wooramel region (Fig. 1). Each of the stations were approximately 120–650 km apart.

As part of the standard management practice, goats were captured by trapping in yards and supplied with oaten hay and water ad libitum. Goats were loaded in the morning (05:30 am) and transported by truck in open trailers for approximately 12 hours to the abattoir in the Beaufort River region. On arrival at the abattoir, goats were kept in a shaded pen with access to water (lairage). Slaughter commenced at 10:00 am on the day following transport.

From each of the four consignments, 100 goats were sampled with specimens collected at regular time intervals (approximately two to four minutes) as the consignment was processed so as to spread the sampling across the entire consignment. After evisceration, goats’ digestive tracts were separated from carcasses for collection of offal. The digesta sample from the caecum was collected using sterile technique as described^6^. Briefly, for each sample, approximately 25 g of faecal contents was obtained by making a transverse incision of the large intestine 10 to 20 cm proximal to the anus using a sterile scalpel blade and then using a sterile-gloved hand to express faecal matter into a sterile polypropylene container. Samples were then immediately labelled, stored on ice or in a refrigerator (4.0 °C), while being transported to the laboratory for isolation of *Salmonella* later that day.

Sample collection methods were approved by Murdoch University Animal Ethics Committee (approval number R2617/13). All methods were carried out in accordance with relevant guidelines and regulations outlined by Murdoch University Animal Ethics Committee.

### *Salmonella* isolation and identification

Samples for isolation of *Salmonella* were as described by Garcia^19^. Briefly, 10 g of each faecal sample were transferred aseptically to each of 7 ml of 0.1% Buffered Peptone Water (BPW) and 7 ml Rappaport–Vassiliadis (RV) broth, mixed, allowed to settle and incubated for 18–24 h at 37 °C and 42 °C, respectively. After incubation, a loopful of each enrichment broth was inoculated onto Xylose Lysine Deoxycholate (XLD) (Oxoid CM0469, Basingstoke, England) agar and Brilliance *Salmonella* (BS) agar (Thermofisher Scientific, Australia) and each were incubated at 37 °C for 24 hrs. Typical *Salmonella* colonies identified by colony morphology (e.g. black and pink colonies on XLD and BS agars respectively) were further cultured into Colombia sheep blood agar (ThermoFisher Scientific, Australia) and incubated at 37 °C for 24 h. From each plate 10–25 colonies were harvested, deposited in Brain Heart Infusion (BHI) broth containing glycerol (20% v/v) and stored frozen at −80 °C until further use.

Identification of organisms as *Salmonella* was achieved using Matrix Assisted Laser Deionised Time of Flight (MALDI–TOF) analysis as per manufacturer’s recommendation. Briefly, all samples of presumptively positive colonies were inoculated onto Colombia sheep blood agar (ThermoFisher Scientific, Australia), incubated at 37 °C for 24 h and then transferred to a target plate (96 MSP, Bruker® – Billerica, USA). One bacterial colony was transferred to the centre of each well of the microplate to avoid cross contamination. Three control wells (without bacteria) were also used on each target plate. The bacterial spot was covered with a lysis solution (70% formic acid; Sigma–Aldrich®) followed by 1 μL aliquot of matrix solution (alpha-ciano-4-hidroxi-cinamic acid diluted in 50% acetonitrile and 2.5% trifluoroacetic acid, Sigma–Aldrich®). The spectra of each sample were collected in a mass range between 2000 and 20,000 m/s, and then were analyzed by the MALDI Biotyper 2.0 (Bruker®) program, using the standard configuration for bacteria identification, which compared the spectrum of the samples with the references in the database. The results vary on a 0–3 scale, where the highest value indicates a more precise match and reliable identification. In the current study, the values accepted for matching were greater than or equal to 2.

### DNA extraction

All the *Salmonella* isolates were sub-cultured on Columbia sheep blood agar (ThermoFisher Scientific, Australia) and incubated overnight at 37 °C. The genomic DNA was purified from a single colony from the overnight cultures using the DNeasy® Blood & Tissue Kit (Qiagen, Hilden, Germany) following the manufacturer’s recommendations and stored at −20 °C until use. A negative control (saline) was used in each extraction group consisting of 24–48 samples performed on the same day.

### PCR amplification and sequencing

All MALDI-TOF positive isolates were subjected to PCR using serovar Typhimurium specific primers targeting the putative hexulose-6-phosphate synthase (STM2755; 406 bp amplicon) and cytoplasmic proteins (STM4497; 523 bp amplicon) and PCR conditions previously described^20^. A previously characterized *S*. Typhimurium isolate [10] was used as a positive control. Isolates which returned negative results using the STM2755 and STM4497 primer sets were further subjected to PCR for the *S*. *enterica* omp*F* (578 bp amplicon) and inv*A* (521 bp amplicon) genes as described^21,22^. Both inv*A* and omp*F* genes are virulence genes located on the *Salmonella* pathogenicity island 1 (SPI 1), that allow rapid molecular detection of *Salmonella* species^22^.

Previously characterized *S*. *enterica* serovars Dublin, Enteritidis, Hadar and Hato^23^ were used as positive controls. PCR products were separated by gel electrophoresis and purified using an in-house filter tip method previously described^24^. Purified PCR products were sequenced using an ABI Prism Dye Terminator Cycle Sequencing kit (Applied Biosystems). Nucleotide sequences were analysed using Chromas lite version 2.0 (http://www.technelysium.com.au) and aligned with reference sequences from GenBank using Clustal W (http://www.clustalw.genome.jp).

### *Salmonella* antimicrobial susceptibility testing and interpretation

Each isolate was subjected to broth microdilution assays (Sensititre; Thermo Fisher Scientific, Waltham, MA) to determine the minimum inhibitory concentrations (MICs) of 13 antimicrobial agents: cefoxitin (CEF), azithromycin (AZI), chloramphenicol (CHL), tetracycline (TET), ceftriaxone (CTX), amoxicillin-clavulanic acid (AMC), ciprofloxacin (CIP), gentamicin (GEN), ceftiofur (CFT), trimethoprim-sulfamethoxazole (TRI), ampicillin (AMP), nalidixic acid (NAL) and streptomycin (STR) against *Salmonella*. MIC results were interpreted as resistant (R), susceptible (S) and intermediate, according to veterinary specific and human approved interpretative criteria as per the Clinical and Laboratory Standards Institute (CLSI) VET01S guidelines^25^ (CLSI, 2015). When clinical breakpoints were not available in CLSI, MICs were interpreted based on epidemiological cut-off values (ECOFFs) as non-wild type (non-WT) organisms derived from assessment of the MIC distribution using ECOFFinder^26,27^ and/or as published by the European Committee on Antimicrobial Susceptibility Testing (EUCAST)^28^ as presented in Table 1. For phenotypic analysis, if ECOFF was not present, the clinical break points were used. *Staphylococcus aureus* ATCC 25923 and ATCC 29213 and *Escherichia coli* ATCC 25922 were used as control strains. *Salmonella* isolates showing clinical resistance to three or more classes of antimicrobial agents were classified as multi-drug resistant (MDR).

**Table 1:** Breakpoints used for susceptibility testing of *Salmonella* species.

Class	Agent	Range (mg/L)	nWT^a^	Clinical Breakpoint^b^	
				CS	CR
Aminoglycosides	Gentamicin	0.25–16	2	≤4	>8
	Streptomycin	2–64	16	≤32	>32
β-lactam/β-lactam inhibitor combination	Amoxicillin-Clavulanate (2:1 ratio)	1–32	—	≤8	>16
Cephems	Cefoxitin	0.5–32	8	≤8	>16
	Ceftiofur	0.12–8	2	—	—
	Ceftriaxone	0.25–64	—	≤1	≥4
Fluoroquinolones	Ciprofloxacin	0.015–4	0.06	≤0.06	>0.5
Quinolones	Nalidixic acid	0.5–16	—	—	—
Folate pathway inhibitors	Trimethoprim-Sulfamethoxazole (1:19)	0.12–4	1	≤2	>2
Macrolides	Azithromycin	0.12–16	—	—	—
Penicillins	Ampicillin	1–32	8	≤8	>16
Phenicols	Chloramphenicol	2–32	16	≤8	>16
Tetracyclines	Tetracycline	4–32	8	≤4	>8

^a^nWT = non-wild type [EUCAST epidemiological cut-off values (mg/L) (ECOFF)].

^b^CLSI VETO1S,(CLSI, 2015) or M100S(CLSI, 2016) breakpoints (mg/L), CS = Clinically-sensitive; CI = Clinically-Intermediate (between CS and CR, not shown); CR = Clinically-resistant.

“—” Not defined.

### Statistical analyses

Statistical analyses were performed using Stata/MP 14.0 (Stata Corp., College Station, TX, USA). Point prevalence was determined by proportion of *Salmonella*-positive goats for each consignment. Prevalence 95% confidence intervals (CI) were calculated using Jeffrey’s interval method^29^. Percentages of AMR and MDR were calculated based on the overall percentage of positive isolates in all examined consignments.

## Results

### Prevalence of *Salmonella*

The overall rate of detection of *Salmonella* faecal carriage was 106/400 (26.5%), with faecal carriage in the four consignments ranging between 23–30% (Table 2). When tested by MALDI-TOF, all *Salmonella* isolates from rangeland goats returned matches to *Salmonella* with values equal or greater than 2, confirming their classification.

**Table 2:** Percent of rangeland goats (*n* = 400) with *Salmonella enteric*a detected in faeces at slaughter in Western Australia showing breakdown by origin of consignment and serovar detected.

Consignment	Percent of goats with *S*. *enterica* detected in faeces (95% CI)			
	All serovars	Serovars		
		*S*. Typhimurium	*S*. Chester	*S*. Saintpaul
Overall (*n* = 400)	26.5 (22.4–31.0)	22.5 (18.6–26.8)	2.8 (1.5–4.7)	1.25 (0.5–2.7)
Carnarvon (*n* = 100)	28 (19.9–37.3)	24 (16.5–33.0)	2 (0.4–6.3)	2 (0.4–6.3)
Shark Bay (*n* = 100)	30 (21.7–39.5)	23 (15.6–31.9)	5 (1.9–10.6)	2 (0.4–6.3)
Yalgoo (*n* = 100)	23 (15.6–31.9)	21 (13.9–29.7)	2 (0.4–6.3)	0 (0.0–2.5)
Wooramel (*n* = 100)	25 (17.3–34.1)	22 (14.8–30.8)	2 (0.4–6.3)	1 (0.1–4.6)

Antimicrobial resistance.

### Molecular typing

*S*. Typhimurium was detected in 90/106 (84.9%) isolates by amplification using serovar specific primers; STM2755 and STM4497. Of the 90 *S*. Typhimurium samples detected, randomly selected subset of isolates (n = 32 isolates; n = 8 isolates per consignment; Overall 26.5% isolates) were sequenced at the STM2755 and STM4497 loci to confirm the molecular typing of the *Salmonella* serovars. Sequencing indicated that *S*. Typhimurium from rangeland goats shared identical genetic similarity with *S*. Typhimurium (CP019649) previously obtained from Australian pigs^30^ (Table 2). The remaining non-typhi isolates 16/106 were amplified and sequenced at invA and ompF loci with 11/106 (10.4%) isolates identified as *S*. Chester (99% homology to GenBank isolate CP019178) and 5/106 (4.7%) isolates identified as *S*. Saintpaul (99% homology to GenBank isolate CP017727).

### Antimicrobial Resistance Characterisation

The majority of isolates (89/106; 84.0%) remained phenotypically susceptible to all 13 antimicrobials in the study (Table 3). Isolates with AMR to one (3/106; 2.9%), two (10/106; 9.4%) and three (4/106; 3.7%) antimicrobials drugs were identified (Table 3), with the four isolates clinically resistant to three classes (β–lactamase, Macrolides and Tetracylines) of the antimicrobial agents classified as multi-drug resistant (MDR). No isolates were resistant to four or more antimicrobials, and none were resistant to either ceftiofur, ceftriaxone, chloramphenicol or ciprofloxacin (Table 3). However, the data shown in Table 3 for amoxicillin-clavulanate, azithromycin and ceftriaxone, represents the percent of non-susceptible isolates due to a lack of breakpoint for wild type. Resistance was most frequently detected to azithromycin (14.2%), followed by tetracycline (10.5%), ampicillin (5.7%), amoxicillin–clavulanate and cefoxitin (3.8%), trimethoprim/sulfamethoxazole (1.9%), gentamicin (0.9%) and streptomycin (0.9%) (Table 3).

**Table 3:** Distribution of MICs and resistance among *Salmonella* isolates (*n* = 106) from faecal samples collected from rangeland goats at slaughter in Western Australia.

Antimicrobial	0.016	0.03	0.06	0.13	0.25	0.5	1	2	4	8	16	32	64	% nWT (95% CI)	% CR 95% CI
Amoxicillin-Clavulanate	—	—	—	—	—	—	89.6	1.9	3.8	0.9			3.8	x	3.8 (1–9.4)
Ampicillin	—	—	—	—	—	—	83	9.4	1.9		0.9	0.9	3.8	5.7 (2.1–11.9)	4.7 (1.5–10.7)
Azithromycin	—	—	—					2.8	47.2	35.8		14.2	—	x	14.2 (8.1–22.3)
Cefoxitin	—	—	—	—	—		0.9	51.9	38.7	4.7	0.9		2.8	3.8 (1–9.4)	2.8 (0.6–8)
Ceftiofur	—	—	—			9.4	85.8	4.7			—	—	—	0 (0–3.4)	—
Chloramphenicol	—	—	—	—	—	—	—		4.7	88.7	6.6		—	0 (0–3.4)	0 (0–3.4)
Ciprofloxacin	22.6	73.6	3.8							—	—	—	—	0 (0–3.4)	0 (0–3.4)
Ceftriaxone	—	—	—	—	95.3	4.7								x	0 (0–3.4)
Gentamicin	—	—	—	—	11.3	75.5	12.3		0.9			—	—	0.9 (0–5.1)	0 (0–3.4)
Naladixic Acid	—	—	—	—	—			4.7	82.1	11.3	1.9		—	x	—
Streptomycin	—	—	—	—	—	—	—	0.9	13.2	56.6	28.3	0.9		0.9 (0–5.1)	—
Tetracycline	—	—	—	—	—	—	—	—	89.6				10.4	10.4 (5.3–17.8)	10.4 (5.3–17.8)
Trimethoprim/Sulfamethoxazole	—	—	—	89.6	7.5		0.9	0.9		0.9	—	—	—	1.9 (0.2–6.6)	0.9 (0–5.1)

nWT, non-wild type; CR, clinical resistance; “x” No data presented for this drug due to lack of wild, susceptible and clinical breakpoints. Cells not denoted as ‘—’ indicate MIC range available for each agent available on the Sensititre CMV3AGNF card. MICs > than highest concentration available are indicated in the shaded region. Thin vertical lines indicate EUCAST ECOFF values and Thick vertical lines indicate CLSI resistant breakpoints.

Resistance was not evenly distributed amongst the *Salmonella* from different consignments of goats: the *Salmonella* from Carnarvon region goats were susceptible to all tested antimicrobials, unlike the other regions (Supplementary Table 1). The only resistant isolates (16.0%; 17/106) were those of *S*. Typhimurium, when typed using primers of putative hexulose-6-phosphate synthase (STM2755) and cytoplasmic proteins (STM4497).

## Discussion

The present study represents the first comprehensive description of AMR from Australian goats, due to the high number of faecal samples tested, and the sparse literature currently available on the subject. High rates (26.5%) of detection of *Salmonella* faecal carriage, but markedly low rates of AMR and MDR were observed in this study. Overall 84% of *Salmonella* isolates from rangeland goats were entirely susceptible to all the antimicrobials tested, and no CIA resistance (fluoroquinolones and third generation cephalosporins) was observed. The majority of the AMR observed was for goats from Yalgoo, and further characterisation of *Salmonella* for this region is warranted. The AMR detection rates for goats were comparable with those reported by Barlow *et al*.^6^ for Australian beef cattle where resistance to tetracycline (6.6%), ampicillin (7.5%), trimethoprim/sulfamethoxazole and streptomycin was observed^6^. This study reported 3.8% cefoxitin-resistance in *Salmonella* from goats for the first time. Cefoxitin resistance in *E*. *coli* has been reported in 14.3% of Australian porcine isolates^5^.

The present study also highlights that antimicrobial resistance to low importance antimicrobials (first line) such as tetracycline and ampicillin can occur in remote animal production system where there is negligible use of antimicrobials. The most likely explanation is that the forms of resistance detected were likely to have been introduced into these regions by humans, animals and/or wild birds^3^. Watering points represent sites where *Salmonella* has potential to persist, and may play a role in transmission to livestock, including goats.

There has been a world-wide increase in the reporting of MDR forms of *Salmonella* isolates from food-producing animals to the level where it represents a risk to human health^31,32^. In Africa and the Indian sub-continent, goats have been reported to contribute to *Salmonella* contamination in the food chain with MDR being present including resistance against CIAs^8,33–35^. In contrast, the results of this study demonstrate low proportions of *Salmonella* isolates expressing AMR and MDR are in agreement with studies reported for other Australian livestock^5,6^. Multiple antimicrobial resistances (mainly streptomycin and tetracycline) were reported in *Salmonella* isolated from Australian dairy cattle^36^ and more recently, resistance to ceftiofur has also been detected^37^. Similarly, 0.6–1.2% of clinical *Salmonella* isolates from pigs showed resistance to trimethoprim, gentamicin and tetracycline^5^.

The rate of detection of *Salmonella* faecal carriage at slaughter (26%) was consistent with a previous study that observed 30% *S*. *enterica* faecal carriage detection in rangeland goats on arrival at a feedlot^18^. Published studies generally report high rates of isolation of *Salmonella* from caprine gut contents and faeces. For example, 46.3% and 45.5% of faecal and rumen samples respectively contained *Salmonella* when 121 rangeland goats were sampled at two Queenslander abattoirs^14^. Rates of detection for *S*. *enterica* from goats at slaughter have also been reported in Nigeria (24.2%) and Ethiopia (3–11.5%)^33,38–41^. Generally, high rates of *Salmonella* detection can be explained by the ruminant digestive tract’s sensitivity to the effects that fasting and ration change have on the presence of *Salmonella*^42^. Thus, factors in this study such as geographic location of origin which impacts on the duration of fasting and also the extent of diet change (since animals are from natural pastures) are all likely reasons why a high proportion of goats were positive for faecal carriage of *Salmonella*.

Agglutination-based serotyping has been the predominant technique for laboratory-based surveillance of *Salmonella* including typing and serovar discrimination. However, molecular typing of *Salmonella* serovars, as demonstrated in this work, can address substantial limitations of the conventional approach with respect to sensitivity and demands on technician time. A further advantage of the molecular approach is that it can identify and differentiate many of those *Salmonella* serovars that do not express O-antigens and/or both phase 1 and 2 flagellar antigens^43–45^. Moreover, molecular serotyping methods are much more accessible since they are not reliant on stocks of agglutination sera and thus are now very attractive for use in conjunction with single bacterial colony isolation for better sensitivity and outbreak investigation.

The three *S*. *enterica* serovars identified in the present study (Typhimurium, Chester and Saintpaul) have all been previously reported in Australian rangeland goats^14^, and the dominance of the broad host range serovar Typhimurium in the present study concurs with a previous study in Western Australia^18^. The *S*. *enterica* serovars identified in the present study have also been reported in Australian feral pigs^46^, wildlife^47^, wild birds^9^, and cattle^6^ indicating all these species and rangeland goats are likely elements in the ecology of *S*. *enterica* that involves multi-directional transmission. A recent study has proposed that water sources contaminated by the faeces of feral pigs could account for the occurrence of *Salmonella* infection in co-grazing animals in remote arid and semi-arid regions^48^ although due to the broad host range of some serovars (e.g. Typhimurium) the true picture is perhaps more complex and involving multiple reservoirs. Thus, ecological studies to determine the role of invasive species in livestock and wildlife in *Salmonella* transmission in rangeland regions are warranted and are now possible with the aforementioned availability of molecular serotyping.

There were some limitations of the present study that should be addressed in future investigations. Future studies will ideally include whole genome sequencing (WGS) of the *Salmonella* isolates to determine the genotypic characteristics of *Salmonella* including virulence determinants, carriage of AMR genes and bacterial serotypes. Notwithstanding this, the findings from the present study demonstrate high faecal carriage of *Salmonella* in slaughtered rangeland goats but with encouragingly low levels of antimicrobial resistance.

## Conclusion

This is the first comprehensive description of antimicrobial resistance in *S*. *enterica* in rangeland goats in Australia. This study demonstrated that goats at slaughter had a high rate of faecal carriage for *Salmonella* spp., but low rates of AMR and MDR detection. High rates of carriage of *Salmonella* spp. are associated with risk of the organism entering the food chain. Further studies are needed to elucidate practical and affordable interventions for reducing the faecal carriage of *Salmonella* at the time feral goats are slaughtered. The work also serves as a prelude for applying whole genome analysis to understand what relationships, if any, exist between caprine-*Salmonella* and those *Salmonella* found in other animals and humans.

## Electronic supplementary material

Dataset 1
