# Supplementary material for: Salmonella enterica isolates from Western Australian rangeland goats remain susceptible to critically important antimicrobials
Source: Sci Rep. 2018 Oct 17;8:15326. doi: 10.1038/s41598-018-33220-5 (PMC6193037; doi:10.1038/s41598-018-33220-5)
Supplement: Supplementary file 1 — Dataset 1 [file 41598_2018_33220_MOESM1_ESM.pdf]

***Salmonella enterica* isolates from Western Australian rangeland goats remain susceptible to critically important antimicrobials**

Khalid Al-Habsi[1], David Jordan[2], Ali Harb[1], Tanya Laird[1], Rongchang Yang[1], Mark O'Dea[1], Caroline Jacobson[1], David W. Miller[1], Una Ryan[1] & Sam Abraham[1]

[1]School of Veterinary and Life Sciences, Murdoch University, Murdoch, WA 6150, Australia. [2]New South Wales Department of Primary Industries, 1243 Bruxner Highway, Wollongbar, NSW 2477, Australia. Correspondence should be addressed to S.A.

(email: S.Abraham@murdoch.edu.au)

Supplementary Table 1. Distribution of MICs and resistance among *Salmonella* isolates (n=106) from faecal samples collected from rangeland goats at slaughter in Western Australia.

Class	Antimicrobial agent	Group [b]	n	% Non-Wild-Type (95% CI)	% Clinical Resistance (95% CI)	Antimicrobial concentration (μg/ml)[a]													
						0.016	0.06	0.06	0.13	0.25	0.5	1	2	4	8	16	32	64	128
β–lactams–β–lactamase inhibitor	Amoxicillin–Clavulanate*	A	28	0.0 (0–12.3)	0.0 (0–12.3)							92.9	3.6	3.6					
		B	30	0.0 (0–11.6)	0.0 (0–11.6)							96.7		3.3					
		C	23	17.4 (5–38.8)	17.4 (5–38.8)							78.3		4.3				17.4	
		D	25	0.0 (0–13.7)	0.0 (0–13.7)							88	4	4	4				
Penicillins	Ampicillin	A	28	0.0 (0–13.7)	0.0 (0–12.3)							92.9	7.1						
		B	30	0.0 (0–11.6)	0.0 (0–11.6)							83.3	13.3	3.3					
		C	23	17.4 (5–38.8)	17.4 (5–38.8)							82.6					4.3	13	
		D	25	8.0 (1–26)	4.0 (0.1–20.4)							72	16	4		4		4	
Macrolides	Azithromycin^	A	28	NA	0.0 (0–12.3)									64.3	35.7				
		B	30	NA	10.0 (2.1–26.5)								10	43.3	36.7		10		
		C	23	NA	30.4 (13.2–52.9)									47.8	21.7		30.4		
		D	25	NA	20.0 (6.8–40.7)									32	48		20		

Cephems–second generation	Cefoxitin	A	28	0.0 (0–12.3)	0.0 (0–12.3)								53.6	32.1	14.3				
		B	30	0.0 (0–11.6)	0.0 (0–11.6)								56.7	43.3					
		C	23	17.4 (5–38.8)	13.0 (2.8–33.6)							4.3	34.8	43.5		4.3		13	
		D	25	0.0 (0–13.7)	0.0 (0–13.7)								60	36	4				
Cephems–third generation	Ceftiofur	A	28	0.0 (0–12.3)	0.0 (0–12.3)						14.3	78.6	7.1						
		B	30	0.0 (0–11.6)	0.0 (0–11.6)						10	86.7	3.3						
		C	23	0.0 (0–14.8)	0.0 (0–14.8)							91.3	8.7						
		D	25	0.0 (0–13.7)	0.0 (0–13.7)						12	88							
	Ceftriaxone*	A	28	0.0 (0–11.6)	0.0 (0–11.6)					92.9	7.1								
		B	30	0.0 (0–14.8)	0.0 (0–14.8)					100									
		C	23	0.0 (0–13.7)	0.0 (0–13.7)					91.3	8.7								
		D	25	0.0 (0–11.6)	0.0 (0–11.6)					96	4								
Phenicols	Chloramphenicol	A	28	0.0 (0–11.6)	0.0 (0–11.6)										96.4	3.6			
		B	30	0.0 (0–14.8)	0.0 (0–14.8)									3.3	93.3	3.3			
		C	23	0.0 (0–13.7)	0.0 (0–13.7)									8.7	82.6	8.7			
		D	25	0.0 (0–11.6)	0.0 (0–11.6)									8	80	12			

Quinolones	Ciprofloxacin	A	28	0.0 (0–12.3)	0.0 (0–12.3)	25	75												
		B	30	0.0 (0–11.6)	0.0 (0–11.6)	30	63.3	6.7											
		C	23	0.0 (0–14.8)	0.0 (0–14.8)	21.7	73.9	4.3											
		D	25	0.0 (0–13.7)	0.0 (0–13.7)	12	84	4											
Aminoglycosides	Gentamicin	A	28	0.0 (0–11.6)	0.0 (0–12.3)					10.7	89.3								
		B	30	0.0 (0–11.6)	0.0 (0–11.6)					3.3	80	13.3		3.3					
		C	23	3.3 (0.1–17.2)	0.0 (0–14.8)					17.4	65.2	17.4							
		D	25	0.0 (0–13.7)	0.0 (0–13.7)					16	64	20							
	Naladixic Acid #	A	28	NA	NA								3.6	96.4					
		B	30	NA	NA								3.3	80	16.7				
		C	23	NA	NA								8.7	78.3	13				
		D	25	NA	NA								4	72	16	8			
Aminoglycosides	Streptomycin	A	28	0.0 (0–11.6)	0.0 (0–12.3)								3.6	17.9	39.3	39.3			
		B	30	0.0 (0–14.8)	0.0 (0–11.6)									10	56.7	33.3			
		C	23	0.0 (0–13.7)	0.0 (0–14.8)									17.4	60.9	21.7			
		D	25	4.0 (0.1–20.4)	0.0 (0–13.7)									8	72	16	4		
Tetracylines	Tetracycline	A	28	0.0 (0–11.6)	0.0 (0–11.6)									100					

		B	30	10.0 (2.1–26.5)	10.0 (2.1–26.5)									90				10
		C	23	17.4 (5.0–38.8)	17.4 (5.0–38.8)									82.6				17.4
		D	25	16.0 (4.5–36.1)	16.0 (4.5–36.1)									84				16
Folate pathway inhibitors	Trimethoprim–Sulfamethoxazole	A	28	0.0 (0–12.3)	0.0 (0–11.6)				96.4	3.6								
		B	30	0.0 (0–11.6)	0.0 (0–14.8)				86.7	10		3.3						
		C	23	4.3 (0.1–21.9)	0.0 (0–13.7)				87	8.7			4.3					
		D	25	4.0 (0–20.4)	4.0 (0–20.4)				88	8					4			

[a] Thin vertical lines indicate EUCAST ECOFF values and thick vertical red lines indicate CLSI resistant breakpoints. The shaded fields indicate the dilution range tested for each antimicrobial. Values in the unshaded area indicate MICs greater than the highest concentration tested.

[b] Groups denoted by letters; A= Carnarvon, B= Shark Bay, C= Yalgoo, D= Wooramel.

* Data represents the percent non-susceptible due to a lack of breakpoint for wild type.

^ Data represents the percent clinically resistant due to lack of breakpoints for both wild type and susceptible.

[#] No data presented for this antimicrobial agent due to lack of wild, susceptible and clinical breakpoint
